# COVID-19 Pandemic and Food Insecurity Fuel the Mental Health Crisis in Africa

**DOI:** 10.3389/ijph.2023.1606369

**Published:** 2024-01-12

**Authors:** Jule Beck, Anke Koebach, Liliana Abreu, Mekdim Dereje Regassa, Anke Hoeffler, Wolfgang Stojetz, Tilman Brück

**Affiliations:** ^1^ Development Research Group, Department of Politics and Public Administration, University of Konstanz, Konstanz, Germany; ^2^ Leibniz Institute of Vegetable and Ornamental Crops, Großbeeren, Germany; ^3^ ISDC—International Security and Development Center, Berlin, Germany; ^4^ Albrecht Daniel Thaer Institute for Agricultural and Horticultural Sciences, Faculty of Life Sciences, Humboldt University of Berlin, Berlin, Germany

**Keywords:** generalized anxiety disorder, mental health, COVID-19, food insecurity, Africa

## Abstract

**Objective:** Providing country-level estimates for prevalence rates of Generalized Anxiety Disorder (GAD), COVID-19 exposure and food insecurity (FI) and assessing the role of persistent threats to survival—exemplified by exposure to COVID-19 and FI—for the mental health crisis in Africa.

**Methods:** Original phone-based survey data from Mozambique, Sierra Leone, Tanzania and Uganda (12 consecutive cross-sections in 2021; *n* = 23,943) were analyzed to estimate prevalence rates of GAD. Logistic regression models and mediation analysis using structural equation models identify risk and protective factors.

**Results:** The overall prevalence of GAD in 2021 was 23.3%; 40.2% in Mozambique, 17.0% in Sierra Leone, 18.0% in Tanzania, and 19.1% in Uganda. Both COVID-19 exposure (OR_adj._ 1.4; CI 1.3–1.6) and FI (OR_adj_ 3.2; CI 2.7–3.8) are independent and significant predictors of GAD. Thus, the impact of FI on GAD was considerably stronger than that of COVID-19 exposure.

**Conclusion:** Persistent threats to survival play a substantial role for mental health, specifically GAD. High anxiety prevalence in the population requires programs to reduce violence and enhance social support. Even during a pandemic, addressing FI as a key driver of GAD should be prioritized by policymakers.

## Introduction

Mental and behavioral illnesses account for 4.9% of disability-adjusted life-years (DALYs); specifically, Generalized Anxiety Disorder (GAD) is amongst the top ten causes of years lived with disability (YLD) [1]. Introduced in the Diagnostic and Statistical Manual (DSM) Version III, GAD is characterized by excessive, uncontrollable worry with a minimum duration of 6 months [2]. Comorbidity is most frequent with depression [3]. Evidence-based treatments such as cognitive behavior therapy and SSRI/SNRI agents are successful in about 50% of cases [3].

Globally, prevalence rates of GAD are estimated at about 0.1%–3.0% (30 days prevalence) in the general population [4]. Multinational studies and a meta-analysis indicate lower GAD rates in low- and middle-income countries [4]. For this study, we conducted a rapid systematic review following PICOS [5] to obtain insights on GAD prevalence rates in the general population in Africa. Presented in Table 1, we found nine studies revealing rates from 0% to 49.6% (see Supplementary Material SI). A part of the variance is due to different assessment methodologies and diagnostic criteria, however recent studies relied predominantly on the GAD-7 of the Patient Health Screening [16]; within these studies the prevalence rates still range from 5.6% in Libya to 49.6% in Nigeria, notably with differences in cut-off levels. Many Africans who suffer from mental illness do not have access to treatment. Treatment gap estimates based on the mere availability of a psychiatrist/clinical psychologist range between 75% and 99% [17, 18].

**TABLE 1:** Studies on prevalence rates of generalized anxiety disorder in the adult general population, country level. (selected studies - Africa, published between 1980 and 2023).

Author(s)	Country	Year of data collection	*N*	Sampling	Prevalence type	Cut-off point	Administration mode	Assessment tool		Prevalence rate
Agberotimi et al. (2020) [6]	Nigeria	2020^a^	502	Snowball	2 weeks	≥5	Online self-report	GAD-7^b^	DSM-5	**49.6%** (vs. 58.4% in healthcare professionals)
Ayazi et al. (2014) [7]	South Sudan	2010	1,200	Multistage random cluster		n.a.^c^	Structured interview	MINI^d^	DSM-4	**15.8%**
Boateng et al. (2021) [8]	Ghana	2020^a^	811	Convenience	2 weeks	≥10	Online self-report	GAD-7	DSM-4	**23.1%**
Bhagwanjee et al. (1998) [9]	South Africa	n.a.	81	Multistage random cluster	Point	≥8	Self-report/structured interview	SRQ-20^e^	DSM-4	**3.7%**
Elhadi et al. (2022) [10]	Libya	2020^a^	31,557	Convenience	2 weeks	≥15	Online/paper self-report	GAD-7	DSM-5	**5.6%**
Gureje et al. (2006) [11]	Nigeria	2001–2003	4,984	Multistage stratified cluster	Lifetime/12 months	n.a.	Structured interview	CIDI^f^	DSM-4	**0.1%/0.0%**
Hollifield et al. (1990) [12]	Lesotho	1986–87	356	Random	1 month	n.a.	Structured interview	NIMH DIS^g^	DSM-3	**6.2%** (with DSM-III hierarchy, vs. 12.9% without)
Jenkins et al. (2015) [13]	Kenya	2013	1,157	Multistage random cluster	1 week	n.a.	Structured interview	CIS-R^h^	ICD-10	**1.6%**
Matsungo and Chopera (2020) [14]	Zimbabwe	2020^a^	507	Convenience	2 weeks	≥10	Online self-report	GAD-7	DSM-5	**40.4%**
Suliman et al. (2010) [15]	South Africa	2002–2004	4,351	Multistage stratified random	12 months	n.a.	Structured interview	CIDI	DSM-4	**1.9%**

Bold values indicate prevalence rates for the general population.

^a^ Peri-pandemic.

^b^ GAD-7, Generalized Anxiety Disorder scale 7 items.

^c^ Not applicable.

^d^ Mini International Neuropsychiatric Interview.

^e^ Self-Reporting Questionnaire 20 items.

^f^ Composite International Diagnostic Interview.

^g^ National Institute of Mental Health Diagnostic Interview Schedule (United States).

^h^ Clinical Interview Schedule—Revised.

In addition to the suffering of affected individuals and their families, costs to society and economic development are enormous as mental illness may render individuals dysfunctional across different life domains. Neuroeconomic experiments further indicate the impact of anxiety and GAD specifically on individual decision making and thus highlights its relevance for collective dynamics and processes when incidence surges [19].

Alongside with genetic predisposition [20], female sex, and parental model learning [21], adverse life events and health issues increasing the vulnerability of a person have shown to increase the risk of GAD [22]. Based on results of a 12 years prospective study, Zhang et al. highlighted that both recent and distal adverse life events independently contributed to GAD [23]. The cumulative impact of *immediate* threats to survival—classified as traumatic events—with posttraumatic stress disorder is well established [24]. Like traumatic events, *persistent* threats to survival may accumulate and impact mood and cognition and compromise mental health via an increase of general anxiety levels. In this study, we estimate prevalence rates for GAD and investigate the interplay of concurring persistent threats in Sub-Saharan Africa (SSA).

**FIGURE 1:**
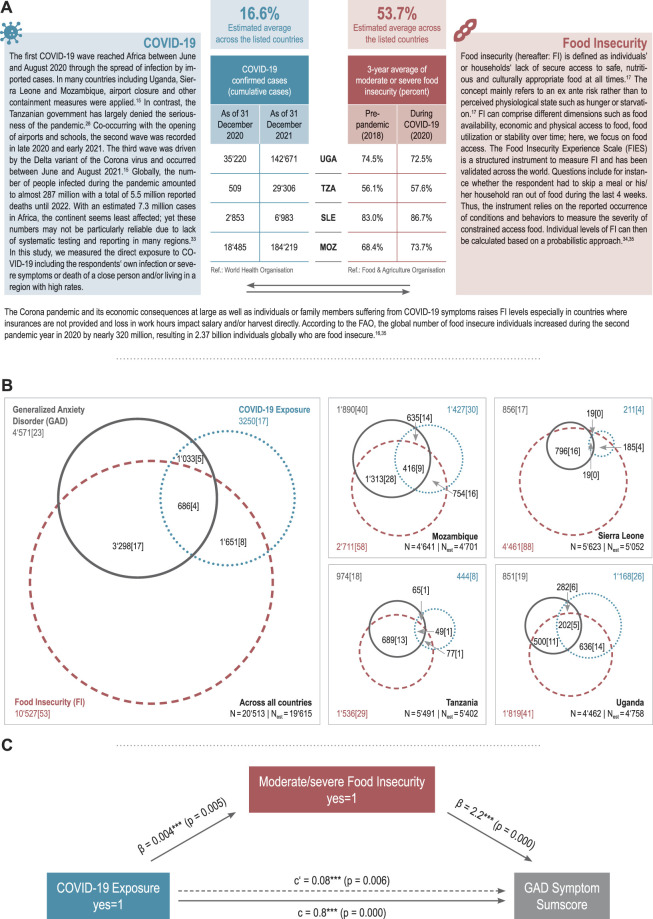
Panel **(A)**: Fact sheet COVID-19 and food insecurity (Life with Corona—Africa, Mozambique, Sierra Leone, Tanzania, Uganda, 2021). Panel **(B)**: Overlap between generalized anxiety disorder, COVID-19 and food insecurity, Venn diagrams (Life with Corona—Africa, Mozambique, Sierra Leone, Tanzania, Uganda, 2021) Panel **(C)**: Determinants of generalized anxiety disorder, mediation model (Life with Corona—Africa, Mozambique, Sierra Leone, Tanzania, Uganda, 2021).

Recently, the mental health crisis in SSA, marked by a surge in the prevalence of mental illness and large treatment gaps, has coincided both with the risk of contracting SARS-CoV-2 [25] and increased levels of food insecurity (FI) [26, 27]. Both the threat of infection and insufficient food insecurity present persistent stressors relevant to survival. Figure 1A provides information about COVID-19 and FI in SSA.

### COVID-19–Anxiety

Globally, the COVID-19 pandemic has caused a surge in anxiety [8, 28]. With a survey covering 58 countries and over 100,000 respondents in March and April 2020, Paudel et al. demonstrated the link between the number of reported COVID-19 cases and anxiety levels [29]. Moreover, containment policies including self-isolation or social distancing orders were shown to be associated with increased anxiety and emotional instability for those who stayed at home [29]. A meta-analysis covering 204 countries pointed to a particularly strong psychological burden of the pandemic for women and younger populations [30]. Studies from the SSA region also indicate a heightened incidence of anxiety during the COVID-19 pandemic [8, 28].

### FI–Anxiety

A recent systematic review of the relationship between FI and mental health in Africa revealed a dose-response relationship [31]. Emerging evidence on the underlying mechanisms suggests that FI directly deteriorates mental health by depriving from fulfillment of a basic need and by creating uncertainty over the ability to meet these needs in the future [32] other mechanisms such as nutrient deficiencies affecting brain function or one’s perceived relative economic status manifested in FI were shown to be of secondary importance [31–33]. The inherent link between FI and mental health is also supported by the study of Jones and colleagues who found a robust association regardless of culture or a countries’ wealth [34].

Our study provides new evidence on the prevalence and associated factors of GAD in the African context. To this end, we draw on novel phone-based survey data providing estimates of prevalence rates of GAD according to the DSM-5 alongside with direct COVID-19 exposure and FI in the general adult population in four African low- and middle-income countries–Mozambique, Sierra Leone, Tanzania and Uganda. We then analyze whether the pandemic adversely affected mental health directly and indirectly by testing whether FI is a mediator of the relation between COVID-19 exposure and GAD.

## Methods

### Study Design and Setting

As part of the Life with Corona (LwC) project [35], the LwC-Africa study collected phone survey data from adults (>17 years) in Mozambique, Sierra Leone, Tanzania and Uganda throughout the year 2021 [36]. During the COVID-19 pandemic, conducting face-to-face interviews could have posed a risk to both the interviewer and the respondent. Moreover, utilizing online surveys in low- and middle-income countries would have introduced a potential bias by selectively including individuals with internet access. To mitigate these challenges, telephone interviews were employed, as a substantial portion of the population possesses a cell phone, offering a more inclusive and accessible mode of data collection. The countries were selected for their variance regarding geography, demographics, and response to the COVID-19 pandemic (e.g., [25, 37]).

### Procedure

The survey was implemented in 12 rounds of data collection from January to December 2021 by a non-governmental organization (BRAC International in Sierra Leone, Tanzania and Uganda) and a local company (Intercampus in Mozambique) using large in-house databases of mobile phone contacts from active and previous research; in each round, participants were randomly selected, but the composition of the population in each country in terms of gender, age, location (urban/rural) was accounted for aiming at 500 new randomly selected participants per month and country. Our sample size was informed by and consistent with the literature [36]. Before the interview, informed oral consent was obtained from all individual participants included in the study by reading out the consent statement at the start of the interviews. The statement contains information regarding the purpose of the interview and the confidentiality of their personal information. The statement also contains information regarding the respondent’s rights to refuse to respond to any individual question or the entire interview. The calls to the respondents were made during the day from the call centers of the survey companies. Usually, two to three calls were made to secure an interview. When repeated attempts to reach a selected respondent were not successful, a replacement satisfying the stratification criteria was used. In line with similar studies [38], the response rate was about 50% whereby the main reasons for non-response were inactive phone contacts and non-response to the phone calls. The average interview duration was 16.5 min. The study was approved by the ethical commission of UNU-WIDER.

### Participants

A total of *N* = 23,943 participants were interviewed (n_Mozambique_ = 6000; n_Sierra Leone_ = 6217; n_Tanzania_ = 6033; n_Uganda_ = 6033). To obtain a representative sample and more precise estimation of prevalence rates per country, we applied post-stratification weights for age, gender and location [39]. Resulting sociodemographic estimates and country comparisons are presented in Table 2.

**TABLE 2:** Socio-demographic characteristics by country (Life with Corona—Africa, Mozambique, Sierra Leone, Tanzania, Uganda, 2021).

	Post-stratified sample (*n* = 24,282)					Unweighted sample (*n* = 24,282)				
	Mozambique (*n = 6,000*)	Sierra Leone (*n = 6,228*)	Tanzania (*n = 6,021*)	Uganda (*n = 6,033*)	*p*-value	Mozambique (*n = 6,000*)	Sierra Leone (*n = 6,228*)	Tanzania (*n = 6,021*)	Uganda (*n = 6,033*)	*p*-value
Female respondent, yes = 1	*(n = 5,999)*	*(n = 6,211)*	*(n = 6,019)*	*(n = 6,031)*		*(n = 6,000)*	*(n = 6,216)*	*(n = 6,021)*	*(n = 6,033)*	
Proportion	52%	51%	51%	51%	0.997	53.8%	58.7%	57.1%	49.9%	0.000
Age of respondent	*(n = 5,999)*	*(n = 6,211)*	*(n = 6,019)*	*(n = 6,031)*		*(n = 6,000)*	*(n = 6211)*	*(n = 6019)*	*(n = 6,031)*	
Mean	32.9	33.3	36.1	33.9	0.000	33.2	36.3	39.1	37.7	0.000
SD^a^	10.1	8.8	12.1	12.3		10.2	8.5	11.16	11.9	
Education of respondent in years	*(n = 5,999)*	*(n = 6,211)*	*(n = 6,019)*	*(n = 6,031)*		*(n = 6,000)*	*(n = 6,216)*	*(n = 6021)*	*(n = 6,033)*	
Mean	11.8	7.0	8.8	9.4	0.000	11.9	8.2	8.8	9.3	0.000
SD	3.3	5.4	2.8	3.6		3.4	5.5	3.10	3.8	
Respondent is married	*(n = 5,999)*	*(n = 6,211)*	*(n = 6,019)*	*(n = 6,031)*		*(n = 6,000)*	*(n = 6,216)*	*(n = 6021)*	*(n = 6,033)*	
Proportion	63.5%	76.4%	62.8%	71.0%	0.000	63.7%	82.6%	66.6%	79.9%	0.000
Number of household members over 60 years	*(n = 5,999)*	*(n = 6,211)*	*(n = 6,019)*	*(n = 6,031)*		*(n = 6,000)*	*(n = 6,216)*	*(n = 6021)*	*(n = 6,033)*	
Mean	0.2	0.6	0.3	0.3	0.195	0.2	0.5	0.3	0.3	0.195
SD	0.5	0.8	0.6	0.6		0.5	0.7	0.6	0.7	
Number of household members under 18 years	*(n = 5,999)*	*(n = 6,211)*	*(n = 6,019)*	*(n = 6,031)*		*(n = 6,000)*	*(n = 6,216)*	*(n = 6,021)*	*(n = 6,033)*	
Mean	2.5	2.8	2.1	3.2	0.000	2.4	2.8	2.00	3.6	0.000
SD	1.8	1.6	1.3	2.2		1.7	1.5	1.4	2.2	
SES^b^ Index	*(n = 5,999)*	*(n = 6,211)*	*(n = 6,019)*	*(n = 6,031)*		*(n = 6,000)*	*(n = 6,216)*	*(n = 6,021)*	*(n = 6,033)*	
Mean	1.0	−1.6	−0.5	−1.0	0.000	1.2	−0.8	0.3	−0.7	0.000
SD	1.6	1.5	1.6	1.3		1.6	1.7	1.6	1.4	
Rural household, yes = 1	*(n = 5,999)*	*(n = 6,211)*	*(n = 6,019)*	*(n = 6,031)*		*(n = 5,999)*	*(n = 6,216)*	*(n = 6,021)*	*(n = 6,033)*	
Proportion	66.0%	59.0%	73.0%	76.0%	0.067	45.8%	14.9%	20.08%	53.8%	0.000

Note: Values are means and standard deviations or proportions. T-tests were used for comparison of continuous variables and Pearson’s χ^2^ tests were used for comparisons of proportions. Number of non-missing values is indicated in brackets.

Italic values indicate the number of non-missing values.

^a^ Standard deviation.

^b^ Socioeconomic status.

### Measures

Computer-assisted telephone interviews were conducted by trained enumerators; the 4 days training included relevant concepts, mock interviews, administration of the computer-assisted telephone interviews (CATI) protocol, piloting of the survey, and debriefing sessions. The original English version of the questionnaire was translated into Luganda, Runyankole, Acholi, Lusoga, Kiswahili, Krio/Creole and Portuguese. Validity of the translations for use in the respective countries was ensured based on repeated mock interviews among the enumerators, fluent speakers of the local languages, during the training as well as the pre-survey piloting of the questionnaire.

*GAD* according to DSM-5 was measured with the 7-item Generalized Anxiety Disorder scale (GAD-7) [16]. Each item (e.g., *Feeling nervous*, *anxious*, or *on edge*; *Not being able to stop or control worrying*; *Worrying too much about different things*) was rated on a scale from 0 (not at all) to 3 (nearly every day) according to the presence of symptoms during the last 2 weeks. A sumscore ranging 0–21 was used to indicate symptom severity. Following a systematic review/meta-analysis conducted by Plummer and colleagues, who presented a sensitivity of 0.83 and specificity 0.84 [40] we used a cut-off value of 8 to estimate the diagnosis of GAD. We present prevalence rates at alternative thresholds in the Supplementary Table S7. Although validation studies are not available for the specific countries included in our study, the GAD-7 is a standardized instrument validated for various (African) countries [41]. Recognizing the importance of context-specific validation, we strongly advocate for further validation studies in African countries to enhance the reliability and applicability of mental health assessment tools in diverse cultural settings.

*COVID-19 exposure* was measured with four dichotomous (yes [1]/no [0]) questions asking whether the respondent was *infected by the Coronavirus, met anyone who acutely suffered from COVID-19 symptoms, felt to live in an area with high incidence or knew someone who died from the consequences of Coronavirus infection*. We had to rely on subjective self-assessment of perceived exposure as there has been a wide gap in testing in most African countries [42]. Similar to other sensitive topics in surveys, data quality regarding questions about COVID-19 exposure may be constrained by survey biases such as reporting bias due to social desirability, fear of stigma and discrimination. For the prevalence rates, we analyzed calculated a score summing up all yes [1] answers; COVID-19 exposure was counted as positive when at least the answer to one of the four questions was yes.

*FI* was measured using the Food Insecurity Experience Scale (FIES) [43]; a globally used and validated measure. The eight questions focus on experiences and behaviors related to difficulties in accessing food (e.g., *worrying about food scarcity, eating less nutritious food, skipping meals or running out of food*) in a 4 weeks recall period and can be answered with yes [1]/no [0]. Using a probabilistic approach, the respondents’ status with regard to a policy relevant level of FI (moderate-or-severe FI) was determined as suggested by FAO [43, 44].

Furthermore, we collected information about sociodemographic characteristics (gender, age, years of education, and marital status), living in a rural/urban area, household composition and wealth indicating the socio-economic status of the respondent (hereafter: SES index); the latter was calculated via Principal Component Analysis to reduce the dimensionality of numerous variables (see Supplementary Material SⅡ). This approach captures household living standards and therefore avoids common measurement biases related to income or expenditure measures [45]; higher SES index indicates more assets, better infrastructure, and housing.

### Statistical Analysis

To estimate prevalence rates of GAD diagnosis and exposure to COVID-19 FI, we use frequencies and percentages on our sample with post-stratification weights applied for age, gender and location (urban/rural). Respondents were excluded when information in any main variable was missing (missing values: n_GAD-7_ = 1; n_COVID-19_ = 3,576; n_FI_ = 230). To examine the association of the pandemic and FI and the risk of developing GAD, we conducted logistic regression analyses. Furthermore, we performed a mediation analysis using structural equation modeling [46] hypothesizing that FI mediates the association of COVID-19 and GAD. To allow for full convergence of the mediation model, we omit country-fixed effects and only include region and survey round dummies. The reliability of mediation analysis relies on certain vital assumptions, including the lack of multicollinearity, minimal measurement errors (especially in the mediator), control of omitted variable bias by incorporating relevant confounders, and linearity. We have found that these necessary assumptions are met, thus confirming the validity of the use of mediation analysis in our study. The statistical analyses were performed in Stata version 17.

## Results

In our post-stratified sample of 20,513 individuals, a total of 23.3% were above the GAD-7-threshold indicating a diagnosis of GAD. By country, 40.2% in Mozambique, 17.0% in Sierra Leone, 18.0% in Tanzania and 19.1% in Uganda suffered from GAD. COVID-19 exposure is highest in Mozambique and Uganda with 30.4% and 26.2%; the proportion of subjects reporting any exposure to COVID-19 is comparably low in Tanzania (8.2%) and Sierra Leone (4.2%). Overall, the estimated prevalence of FI was 53.7%. The estimated prevalence rate of FI is highest in Sierra Leone (88.3%), followed by Mozambique (57.7%), Uganda (40.8%), and Tanzania (28.4%). Venn diagrams (Figure 1B) show a substantial overlap of GAD with both COVID-19 exposure and FI. Supplementary Table S2 presents variation in prevalence rates over time.

Logistic regression models (Table 3) show that both COVID-19 exposure and FI present as significant risk factors of GAD. Being exposed to COVID-19 is associated with 1.7 (CI 1.4–2.0; *p* = 0.000) higher odds of GAD, while being moderate/severe FI is associated with 2.9 higher odds of GAD compared to food secure individuals (CI 2.4–3.4; *p* = 0.000). The adjusted model accounts for respondents’ characteristics, region, country and survey round. The effect of FI on GAD (adj. OR 3.1; CI 2.6–3.7; *p* = 0.000) is more than twice as large as COVID-19 exposure (adj. OR 1.4; CI 1.3–1.6; *p* = 0.000). While each additional young household member (OR 1.1; CI 1.1–1.1; *p* = 0.000) is significantly associated with higher odds of GAD, higher SES presents as protective factor (OR 0.8; CI 0.8–0.9; *p* = 0.000).

**TABLE 3:** Logistic regression of the determinants of generalized anxiety disorder (Life with Corona—Africa, Mozambique, Sierra Leone, Tanzania, Uganda, 2021).

GAD	Unadjusted		Adjusted	
	OR (95% CI)	*p*-value	OR (95% CI)	*p*-value
COVID-19 exposure, yes = 1	1.7 (1.4, 2.0)	0.000	1.4 (1.3, 1.6)	0.000
Moderate/severe food insecurity, yes = 1	2.9 (2.4, 3.4)	0.000	3.1 (2.6, 3.7)	0.000
Female respondent, yes = 1			1.2 (1.0, 1.3)	0.059
Age of respondent			1.0 (1.0, 1.0)	0.260
Education of respondent in years			1.0 (1.0, 1.0)	0.777
Married, yes = 1			0.9 (0.7, 1.0)	0.098
Number of household members over 60 years			1.0 (0.9, 1.1)	0.998
Number of household members under 18 years			1.1 (1.1, 1.1)	0.000
SES index			0.8 (0.8, 0.9)	0.000
Rural household, yes = 1			1.0 (0.9, 1.1)	0.961
Constant	0.1 (0.1, 0.2)	0.000	0.2 (0.1, 0.3)	0.000
Observations	20,513		20,472	

Note: Logistic regression (odds ratios). Post-stratification weights were applied to all estimates; Adjusted model also accounts for region, country and survey round.

The mediation model (Table 4 and Figure 1C) shows a significant total effect of COVID-19 exposure on GAD (*β* = 0.08, *p* = 0.000). It also confirms a significant association between COVID-19 exposure and FI (*β* = 0.04, *p* = 0.005) and a significant association between FI and the GAD symptom sum score (*β* = 2.2, *p* = 0.000). Moreover, a significant direct effect of COVID-19 exposure on the GAD symptom sum score (direct effect: *β* = 0.8, *p* = 0.000) is detected. The mediation model shows a significant indirect (partly mediated) effect of COVID-19 on GAD trough FI (indirect effect: *β* = 0.1, *p* = 0.005). About 9.4% of the effect of COVID-19 on GAD is mediated by FI and the mediated effect is about 0.1 times as large as the direct effect.

**TABLE 4:** Mediation analysis of the determinants of generalized anxiety disorder (Life with Corona—Africa, Mozambique, Sierra Leone, Tanzania, Uganda, 2021).

	*β*	*p*-value
COVID-19 on FI	0.04	0.005
FI on anxiety score	2.2	0.000
COVID-19 on anxiety score	0.8	0.000
Indirect effect (unstandardized) Monte Carlo test	0.08	0.006
RIT (Indirect effect/Total effect)	9.4%	
RID (Indirect effect/Direct effect)	0.1	
Observations	20,513	

Note: Mediation analysis using structural equation modeling (SEM) and Stata package “medsem” [46] following the approach described in Zhao et al. (2010). Post-stratification weights were applied to all estimates; Model is adjusted for respondent’s characteristics, region, and survey round. Dependent variable: anxiety score; Independent variable: COVID-19; Mediator: food insecurity.

## Discussion

This study finds an overall estimated prevalence of 23.3% for GAD in Mozambique, Sierra Leone, Tanzania and Uganda; 8.4% were directly exposed to COVID-19% and 16.4% presented with moderate-to-high levels of FI. 16.8% of individuals with GAD are food insecure and 8.4% have been exposed to COVID-19; 3.5% of the participants were exposed to both persistent stressors. Accordingly, logistic regression shows that exposure to COVID-19 and in particular FI is associated with a risk of developing GAD. A higher number of dependents under 18 years and lower SES also presented as risk factors for GAD whereas gender and other sociodemographic variables did not reach significance. The mediation analysis reveals that FI as a preexisting persistent threat to survival works as an additional vehicle transforming the stress induced by the pandemic into GAD.

Estimated peri-pandemic GAD prevalence rates –40.2% in Mozambique, 17.0% in Sierra Leone, 18.0% in Tanzania and 19.1% in Uganda– assessed with the GAD-7 at a cut-off point ≥8 following Plummer and colleagues [40] reflect a variance of comparable previous studies: In Ghana, Boateng et al. found 23.1% [8] and in Zimbabwe Matsungo et al. found 40.4% [14]; both with a cut-off point at GAD-7 ≥ 10. In Nigeria, Agberotimi and colleagues found 49.6% (GAD-7 ≥ 5) [6]. In a large study of more than 30 k participants and a strict cut-off score GAD-7 ≥ 15, Elhadi and colleagues found 5.6% in Libya [10]. For comparison, Jia et al. conducted a study in United Kingdom and found a prevalence rate of 23.6% (cut off ≥ 8) [47] and Solomou and colleagues found a rate of 23.1% (GAD-7 ≥ 10) in Cyprus [48]. Prevalence rates for our data with the various cut-off scores for each country are presented in the Supplementary Table S7. All studies that assessed GAD during the pandemic applied the GAD-7 online as self-report instrument and were conducted in 2020. For a better understanding of the prevalence rates of GAD in Africa, more rigorous epidemiological and longitudinal research is needed, including validation studies with expert clinical diagnostic interviews. Moreover, whether COVID-19 related GAD cases are in remission as the pandemic declines warrants further research.

Notably, GAD prevalence rates of this study mirror the patterns of confirmed COVID-19 cases as reported by the WHO: The proportion of respondents reporting COVID-19 exposure is comparably low in Tanzania (8.2%) and Sierra Leone (4.2%) versus Uganda (26.2%) and Mozambique (30.4%). Moreover, our estimated rates of FI reflect those reported by FAO and the relative status of countries: The FI rate is highest in Sierra Leone (88.3%), followed by Mozambique (57.7%), Uganda (40.8%), and Tanzania (28.4%). However, except for Mozambique, our FI rates are significantly lower than those of the FAO as discussed in the limitations below.

Primarily and in line with prospective studies [23], we found that both–COVID-19 and FI–independently presented significant risk factors for GAD. Statistical significance is determined using a threshold of *p* < 0.05. Moreover, the direct effect of moderate/severe FI –the preceding persistent threat– is substantively stronger than the direct exposure to COVID-19: OR_Cov-19_ = 1.7 vs. OR_FI_ = 2.9; while the GAD risk doubles in face of COVID-19 exposure, it triples with a moderate/severe level of FI. Whether this is due to its potentially preceding character, or the severity of threat warrants further exploration. Importantly however, our results suggest that moderate/severe FI further emerges as a mediator for the relation of COVID-19 and GAD explaining 9.4% of the total effect of COVID-19 on GAD. Unsurprisingly, the number of dependents (<17 years) presented as a risk factor and higher SES protected from GAD. Interestingly, we did not find an increased risk for women –a risk factor for GAD in many other studies [49]. This may indicate that gender disparity decreases as persistent threats to survival increase; further analyses are however required to understand this aspect.

### Limitations

Acknowledging the limitations inherent in our study, we stress that our analysis can only estimate associations and not infer causal effects due to the cross-sectional nature of the observational data. Moreover, FI prevalence might be underestimated due to the sampling strategy via mobile phones. For the 2019–2021 timeframe, an estimated 79% of the adult population (<17 years) in Uganda own a cell phone, 75% in Tanzania; 76% in Sierra Leone; and 62% in Mozambique [50]; individuals without a mobile phone potentially show higher FI values. Consequently, GAD prevalence rates might also be higher. However, since we control for household composition and wealth, we guard against possible bias in our analyses. Another limitation is that self-administered instruments such as the GAD-7 may not allow for differential diagnosis, but rather provide complementary information. Nevertheless, sensitivity and specificity tests in validation studies show that GAD-7 has acceptable properties for identifying GAD compared to the established gold standard clinical diagnosis [40].

### Conclusion

Our analysis suggests two main insights: *Firstly*, we learn from our data that about 2–4 in ten individuals –a considerable proportion– in Mozambique, Sierra Leone, Tanzania and Uganda presented with excessive and uncontrollable worries during the COVID-19 pandemic. *Secondly*, persistent stressors such as COVID-19 and moderate/severe FI but particularly FI contribute significantly to the risk of GAD. What does this tell us about the African experience of the pandemic, its legacy for development and avenues for policy action?

Anxiety induces the action disposition of avoidance or defense. While fueling anxiety may carry the advantage of stronger compliance to contagion containment measures, e.g., [48, 51], it may also cause higher GAD rates (amongst other consequences): For instance, a meta-analysis with 72,585 participants [52] revealed that participants with GAD presented with a higher incidence of lifetime perpetration of intimate partner violence. Assessing the transgenerational impact of GAD, Woodruff et al. found mothers with high anxiety levels less engaged with their children [53] and Moore et al. found less warmth, and higher levels of control and criticism [54]. Other transgenerational studies consistently indicate high GAD incidence rates in the offspring. Moreover, experimental laboratory studies show that patients with GAD are more likely to accept and react less strongly to unfair decision [55]. Thus, community-level social corrective reactions may decline while actual and subjective safety and stability in the families may decrease. Higher levels of day-to-day violence in communities and families should be considered as immediate consequence alongside with additional waves of migration. This indicates priority areas for peri- and post-pandemic development programs which include the reduction of violence at the family and community level, as well as an increase in social support.

Moreover, our findings underscore the detrimental effects of concurring persistent stressors on GAD risk. In contrast to other risk factors for anxiety disorders highlighted in previous research, such as immediate threats to survival (psychological trauma), genetic predisposition, and/or health status, we broaden the focus to include persistent threats to survival. In this context, the array of possible policy responses expands: Ensuring that all people can meet their basic needs would not only address these long-term stressors but would also have an important multiplier function. Thus, our findings have immediate policy implications for supporting food programs, as food security is a fundamental determinant of resilience and particularly important when other threats to human life arise. Moreover, addressing food insecurity lends itself more readily to population-wide interventions [56]. Thus, even during global health crises such as the COVID-19 pandemic, addressing FI as a key driver of GAD should be a top priority for policymakers.
