# Supplementary material for: COVID-19 Pandemic and Food Insecurity Fuel the Mental Health Crisis in Africa
Source: Int J Public Health. 2024 Jan 12;68:1606369. doi: 10.3389/ijph.2023.1606369 (PMC10811217; doi:10.3389/ijph.2023.1606369)
Supplement: Supplementary file 1 [file DataSheet1.pdf]

International Journal of Public Health (IJPH)

Supplementary material: Covid-19 pandemic and food insecurity fuel the mental health crisis in Africa

List of Tables

Table 1: Data collection calendar 5
Table 2: Number of respondents per round and country 5
Table 3: Measures 7
Table 4: Correlations (with post-stratification weights) 8
Table 5: Socio-demographic characteristics by country (unweighted) 9
Table 6: Prevalence of Generalized Anxiety Disorder (GAD), Covid-19 exposure and food insecurity (unweighted) 9
Table 7: Prevalence of Generalized Anxiety Disorder (GAD) with different thresholds (with post-stratification weights) 9
Table 8: GAD prevalence by Covid-19 exposure, food insecurity and age group 11
Table 9: GAD prevalence by location, gender and age group 11
Table 10: Logistic regression (specifying country effects) 11
Table 11: Logistic regression (excluding observations from Mozambique) 12

List of Figures

Figure 1: PRISMA Flow diagram 4
Figure 2: Prevalence over survey rounds and lean months by country (with post-stratification weights) 10

I. RAPID SYSTEMATIC LITERATURE REVIEW

This rapid systemic literature review seeks to summarize the available studies that assessed prevalence rates of Generalized Anxiety Disorder (GAD) according to the Diagnostic and Statistical Manual – Version or International Classification of Diseases in the general adult population of African countries.

1. Methods

1.1. PICOS Eligibility

Participants/Population
We will consider all studies that include adult (>17 years of age) human subjects and were conducted in Africa.

Interventions - n.a.

Comparison - n.a.

Outcomes
The primary outcome of interest is point, period or lifetime prevalence of GAD diagnosis according to DSM or ICD assessed with a validated instrument via clinical interviews or self-reported.

Studies
We will only consider quantitative observational studies evaluating the prevalence of GAD such as cross-sectional or panel analyses. We will only include studies relying on representative samples (with respect to gender and age).

1.2. Search strategy

The search strategy seeks to identify published and peer-reviewed materials and will contain four stages. Articles published between 1980 and 2023 will be searched in the following databases: PubMed Central and Google Scholar.

1.2.1. Stage 1: Search to identify keywords

A search will be conducted in the PubMed and Google Scholar databases to identify relevant keywords and synonyms.

The initial keywords are:
generalized anxiety disorder /OR generalised anxiety disorder (title or abstract)
AND prevalence/ OR epidemiology (title or abstract)
African country name (title or abstract) --> search separately
Africa (title or abstract or text) --> search separately

1.2.2. Stage 2: Identification

A search will be conducted using the updated list of keywords and criteria.

Databases: PubMed and Scopus

Criteria:

- ☐ ‘human’ studies
- ☐ published between 1980 and 2023

1.2.3. Stage 3: Screening (based on title/abstract)

The papers identified in stage 2 will be screened (based on title/abstract**)** according to following criteria:

Step one: select studies for…

- ☐ Adult population
- ☐ Validated GAD assessment tool (e.g. CIDI, SKID, MINI, GAD-7, etc.; <u>not</u> unstructured interviews)
- ☐ Studies that report the prevalence of anxiety disorders, subthreshold disorders, or isolated anxiety symptoms (sleep problems, etc.) are <u>excluded</u>
- ☐ Prevalence rate including point, period and lifetime

Step two: separate studies

- ☐ General population (gender and age) vs subgroups (e.g. survivors of violence or patients with deliberating physical disease, etc.)

1.2.4. Stage 4: Check eligibility (based on full text)

The papers that are included after the screening in stage 2 will be checked for eligibility (based on the full text**)** according to following criteria:

Inclusion criteria:

- ☐ Full-text available
- ☐ Peer-reviewed
- ☐ English

Exclusion criteria:

- ☐ Grey literature
- ☐ Reviews
- ☐ Editorials
- ☐ Commentaries
- ☐ Letters
- ☐ Other languages

Example search strategy from PubMed

1,"(""Generalised anxiety disorder"" OR ""Generalized anxiety disorder""[Title/Abstract]) AND ""prevalence""[Title/Abstract] AND ((""Africa""[Title/Abstract] OR ""Africa""[Text Word]) OR Burkina Faso[Title/Abstract] OR Burundi[Title/Abstract] OR Cabo Verde[Title/Abstract] OR Cameroon[Title/Abstract] OR Central African Republic[Title/Abstract] OR Chad[Title/Abstract] OR Comoros[Title/Abstract] OR Democratic Republic of the Congo[Title/Abstract] OR Republic of the Congo[Title/Abstract] OR Côte d'Ivoire[Title/Abstract] OR Djibouti[Title/Abstract] OR Egypt[Title/Abstract] OR Equatorial Guinea[Title/Abstract] OR Eritrea[Title/Abstract] OR Eswatini[Title/Abstract] OR Ethiopia[Title/Abstract] OR Gabon[Title/Abstract] OR Gambia[Title/Abstract] OR Ghana[Title/Abstract] OR Guinea[Title/Abstract] OR Guinea-Bissau[Title/Abstract] OR Kenya[Title/Abstract] OR Lesotho[Title/Abstract] OR Liberia[Title/Abstract] OR Libya[Title/Abstract] OR Madagascar[Title/Abstract] OR Malawi[Title/Abstract] OR Mali[Title/Abstract] OR Mauritania[Title/Abstract] OR Mauritius[Title/Abstract] OR Morocco[Title/Abstract] OR Mozambique[Title/Abstract] OR Namibia[Title/Abstract] OR Niger[Title/Abstract] OR Nigeria[Title/Abstract] OR Rwanda[Title/Abstract] OR São Tomé and Príncipe[Title/Abstract] OR Senegal[Title/Abstract] OR Seychelles[Title/Abstract] OR Sierra Leone[Title/Abstract] OR Somalia[Title/Abstract] OR South Africa[Title/Abstract] OR South Sudan[Title/Abstract] OR Sudan[Title/Abstract] OR Tanzania[Title/Abstract] OR Togo[Title/Abstract] OR Tunisia[Title/Abstract] OR Uganda[Title/Abstract] OR Zambia[Title/Abstract] OR Zimbabwe[Title/Abstract])",,,"(""Generalised anxiety disorder""[All Fields] OR ""Generalized anxiety disorder""[Title/Abstract]) AND ""prevalence""[Title/Abstract] AND (""Africa""[Title/Abstract] OR ""Africa""[Text Word] OR ""burkina faso""[Title/Abstract] OR ""Burundi""[Title/Abstract] OR ""cabo verde""[Title/Abstract] OR ""Cameroon""[Title/Abstract] OR ""central african republic""[Title/Abstract] OR ""Chad""[Title/Abstract] OR ""Comoros""[Title/Abstract] OR ""democratic republic of the congo""[Title/Abstract] OR ""republic of the congo""[Title/Abstract] OR ""cote d ivoire""[Title/Abstract] OR ""Djibouti""[Title/Abstract] OR ""Egypt""[Title/Abstract] OR ""equatorial guinea""[Title/Abstract] OR ""Eritrea""[Title/Abstract] OR ""Eswatini""[Title/Abstract] OR ""Ethiopia""[Title/Abstract] OR ""Gabon""[Title/Abstract] OR ""Gambia""[Title/Abstract] OR ""Ghana""[Title/Abstract] OR ""Guinea""[Title/Abstract] OR ""Guinea-Bissau""[Title/Abstract] OR ""Kenya""[Title/Abstract] OR ""Lesotho""[Title/Abstract] OR ""Liberia""[Title/Abstract] OR ""Libya""[Title/Abstract] OR ""Madagascar""[Title/Abstract] OR ""Malawi""[Title/Abstract] OR ""Mali""[Title/Abstract] OR ""Mauritania""[Title/Abstract] OR ""Mauritius""[Title/Abstract] OR ""Morocco""[Title/Abstract] OR ""Mozambique""[Title/Abstract] OR ""Namibia""[Title/Abstract] OR ""Niger""[Title/Abstract] OR ""Nigeria""[Title/Abstract] OR ""Rwanda""[Title/Abstract] OR ""sao tome and principe""[Title/Abstract] OR ""Senegal""[Title/Abstract] OR ""Seychelles""[Title/Abstract] OR ""sierra leone""[Title/Abstract] OR ""Somalia""[Title/Abstract] OR ""south africa""[Title/Abstract] OR ""south sudan""[Title/Abstract] OR ""Sudan""[Title/Abstract] OR ""Tanzania""[Title/Abstract] OR ""Togo""[Title/Abstract] OR ""Tunisia""[Title/Abstract] OR ""Uganda""[Title/Abstract] OR ""Zambia""[Title/Abstract] OR ""Zimbabwe""[Title/Abstract])"

Search for Scopus database is similarly constructed.

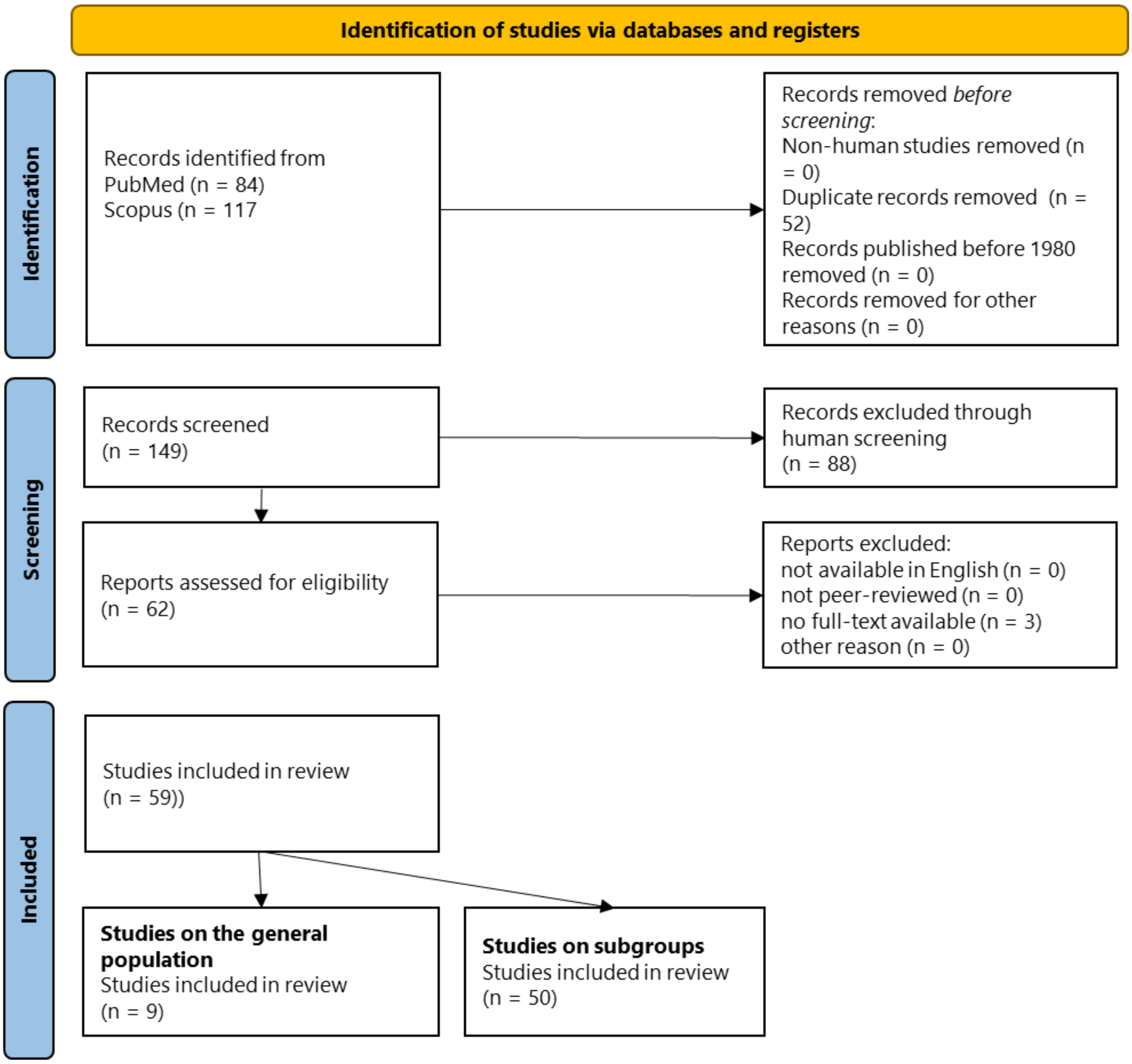

Figure 1: PRISMA Flow diagram

II. STATISTICAL ANALYSIS

Handling of missing data

The number of non-missing values on which the estimates are calculated is given in our respective descriptive statistics. For the logistic regression and mediation models using structural equation modelling, we treat missing data by using listwise deletion, which is the most commonly used method for missing data.

Country	Round											
	1	2	3	4	5	6	7	8	9	10	11	12
**Mozambique**	20 Jan – 08 Feb	10 Feb – 26 Feb	05 Mar – 23 Mar	05 Apr – 21 Apr	05 May – 21 May	07 Jun – 22 Jun	07 Jul – 29 Jul	05 Aug – 23 Aug	06 Sep – 23 Sep	05 Oct – 22 Oct	05 Nov – 22 Nov	01 Dec – 15 Dec
**Sierra Leone**	29 Jan – 08 Feb	26 Feb – 18 Mar	17 Mar – 10 Apr	28 Apr – 06 Jun	31 May – 10 Jun	28 May – 07 Jul	14 Jul – 26 Jul	17 Aug – 27 Aug	21 Sep – 01 Oct	19 Oct – 04 Nov	22 Nov – 13 Dec	08 Dec – 20 Dec
**Tanzania**	27 Dec – 08 Feb	26 Feb – 27 Mar	23 Mar – 27 Mar	28 Apr – 15 May	17 May – 11 Jun	15 Jun – 22 Jun	19 Jul – 26 Jul	13 Aug – 18 Aug	17 Sep – 21 Sep	13 Oct – 18 Oct	18 Oct – 26 Nov	02 Dec – 07 Dec
**Uganda**	06 Jan – 10 Feb	25 Feb – 11 Apr	17 Mar – 01 Apr	26 Apr – 17 May	27 May – 14 Jun	23 Apr – 02 Jul	12 Jul – 26 Jul	09 Aug – 30 Aug	13 Sep – 01 Oct	12 Oct – 31 Oct	11 Nov – 06 Dec	01 Dec – 15 Dec

Table 1: Data collection calendar

Country	Round												
	1	2	3	4	5	6	7	8	9	10	11	12	Total
**Mozambique**	500	500	500	500	500	500	500	500	500	500	500	500	6'000
**Sierra Leone**	506	497	516	527	522	500	516	522	521	520	530	540	6'217
**Tanzania**	489	502	497	508	512	500	504	501	502	502	499	505	6'021
**Uganda**	504	534	483	506	500	500	500	501	504	502	502	497	6'033
**Total**	1'999	2'033	1'996	2'041	2'034	2'000	2'02	2'024	2'027	2'024	2'031	2'042	24'271

Table 2: Number of respondents per round and country

Indicator	Description	Survey questions	Calculation	Validation
**Generalized Anxiety Disorder (GAD)**	binary indicator,<br>1 = GAD	Over the last 14 days, how often have you been bothered by…?<br>A Feeling nervous, anxious or on edge<br>B Not being able to stop or control worrying<br>C Worrying too much about different things<br>D Trouble relaxing<br>E Being so restless that it's hard to sit still<br>E Becoming easily annoyed or irritable<br>F Feeling afraid something awful is going to happen	Not at all: 0<br>Some days: 1<br>More than half days: 2<br>Nearly every day: 3<br>Severity score is the sum of the reported severity score of all questions; Score ranges from 0 to 21 [1]<br>Cut-off value for moderate-or-severe depression is 10; Cut-off value for likely diagnosis of generalized anxiety disorder is 8 as described in Plummer et al. (2016) [2]	Spitzer RL, Kroenke K, Williams, Janet B., Löwe B, Williams JBW. A Brief Measure for Assessing Generalized Anxiety Disorder: The GAD-7. Arch Intern Med. 2006;166(10):1092–7. doi:10.1001/archinte.166.10.1092 Cited in: PubMed; PMID 16717171.
**Food insecurity**	binary indicator,<br>1 = moderate-or-severe food insecurity	During the last 4 weeks, was there a time when – because of lack of money or other resources, you /others in your household…?<br>A Were worried you would not have enough food to eat<br>B Were unable to eat healthy and nutritious food<br>C Ate only a few kinds of foods<br>D Had to skip a meal<br>E Ate less than you thought you should<br>F Household ran out of food<br>G Were hungry but did not eat<br>H Went a whole day without eating	Survey-based experiential measure of access to food<br>A scoring algorithm based on a Rasch model and country-specific thresholds categorizes respondents as food secure, mildly FI, moderately FI or severely FI as described in FAO (2016) [3]	Food and Agriculture Organization of the United Nations (FAO). Methods for estimating comparable prevalence rates of food insecurity experienced by adults throughout the world. Rome; 2016.
**Perceived COVID-19 exposure**	binary indicator,<br>1 = any exposure to COVID-19	Have you ever had, or do you believe that you have ever had, the coronavirus?<br>In the last 14 days, do you think you have met (seen) anyone who you think had the coronavirus when you met them?<br>Do you think your area has a high incidence of coronavirus?<br>Do you personally know someone who has died from the coronavirus in your area?	Indicator takes the value of 1 if any of the four questions is answered with yes	
**Gender**	binary indicator,<br>1 = rural	Gender: Are you?<br>Male: 1<br>Female: 2<br>Other: 3		
**Age**	Age in years	How old are you?		

Indicator	Description	Survey questions	Calculation	Validation
**Married**	binary indicator, 1 = married/cohabiting	Are you currently married/cohabiting?		
**Education**	Education in years	How many years of education have you completed (e.g., in primary school, secondary school, university or vocational skills learning institution)?		
**HH members under 18/over 60**	number of household members	How many of the other people you live with at your household are under the age of 18?<br>How many of the people you live with at your household are over the age of 60 (including yourself)?		
**Socio-economic status (SES)**	index	Do you have access to piped water?<br>Yes (in house): 1<br>Yes (outside the house): 2<br>No: 3<br>Do you have access to electricity in your residence?<br>Yes: 1<br>No: 2<br>How many separate rooms does your house have?<br>Does your household own…?<br>A Radio/Tape recorder<br>B TV<br>C Sofa set<br>D Refrigerator<br>E Car<br>F Bicycle/Motorcycle/Cart<br>G Land/land for farming<br>E Livestock	Asset index was created using a principal component analysis [4] including the following indicators:<br>water in house, water outside house, electricity, rooms, household items	Vyas S, Kumaranayake L. Constructing socio-economic status indices: how to use principal components analysis. Health Policy and Planning. 2006;21459–68.
**Rural**	binary indicator, 1 = rural	Where do you live (place of residence)?<br>In the capital city: 1<br>In urban areas other than the capital city: 2<br>Peri-urban (near a large city or town): 3<br>Rural: 4		

Table 3: Measures

	Likely Diagnosis of GAD, yes=1	Food insecure, yes=1	COVID-19 Exposure	Female respondent, yes=1	Age of respondent	Education of respondent in years	Respondent is married, yes=1	Household nr > 60y	Household nr < 18y	Asset Index	Rural household, yes=1
**GAD**	1.00										
**FI**	0.23***	1.00									
**COVID-19**	0.11***	0.01	1.00								
**Female**	0.03***	0.07***	-0.06***	1.00							
**Age**	-0.03***	-0.11***	0.01	-0.09***	1.00						
**Education**	-0.01	-0.14***	0.12***	-0.22***	-0.23***	1.00					
**Married**	-0.03***	0.00	0.01	-0.13***	0.23***	-0.09***	1.00				
**HH nr > 60y**	0.03***	0.09***	-0.03***	0.03***	0.20***	-0.16***	0.03***	1.00			
**HH nr < 18y**	0.12***	0.10***	0.06***	-0.02***	0.18***	-0.13***	0.21***	0.19***	1.00		
**Asset Index**	-0.02**	-0.26***	0.14***	-0.08***	0.03***	0.40***	-0.03***	-0.08***	-0.08***	1.00	
**Rural**	0.07***	-0.02**	0.02*	-0.00	0.01	-0.07***	-0.00	0.03***	0.12***	-0.22***	1.00

Note: *p < .05, **p < .01, ***p < .001

Table 4: Correlations (with post-stratification weights)

	Mozambique (n=6'000)	Sierra Leone (n=6'228)	Tanzania (n=6'021)	Uganda (n=6'033)	p-value
**Female respondent, yes=1**	*(n=6'000)*	*(n=6216)*	*(n=6021)*	*(n=6033)*	
**Proportion**	53.8%	58.7%	57.1%	49.9%	0.000
**Age of respondent**	*(n=6'000)*	*(n=6211)*	*(n=6019)*	*(n=6031)*	
**Mean**	33.2	36.3	39.1	37.7	0.000
**SD**	10.2	8.5	11.16	11.9	
**Education of respondent in years**	*(n=6000)*	*(n=6216)*	*(n=6021)*	*(n=6033)*	
**Mean**	11.9	8.2	8.8	9.3	0.000
**SD**	3.4	5.5	3.10	3.8	
**Respondent is married**	*(n=6000)*	*(n=6216)*	*(n=6021)*	*(n=6033)*	
**Proportion**	63.7%	82.6%	66.6%	79.9%	0.000
**Nr hh members over 60y**	*(n=6000)*	*(n=6216)*	*(n=6021)*	*(n=6033)*	
**Mean**	0.2	0.5	0.3	0.3	0.195
**SD**	0.5	0.7	0.6	0.7	
**Nr hh members under 18y**	*(n=6000)*	*(n=6216)*	*(n=6021)*	*(n=6033)*	
**Mean**	2.4	2.8	2.00	3.6	0.000
**SD**	1.7	1.5	1.4	2.2	
**SES Index**	*(n=6000)*	*(n=6216)*	*(n=6021)*	*(n=6033)*	
**Mean**	1.2	-0.8	0.3	-0.7	0.000
**SD**	1.6	1.7	1.6	1.4	
**Rural household, yes=1**	*(n=5999)*	*(n=6216)*	*(n=6021)*	*(n=6033)*	
**Proportion**	45.8%	14.9%	20.08%	53.8%	0.000

Note: Values are means and standard deviations or proportions. T-tests were used for comparison of continuous variables and Pearson's χ2 tests were used for comparisons of proportions. Number of non-missing values is indicated in brackets.

Table 5: Socio-demographic characteristics by country (unweighted)

	Mozambique (n=6'000)	Sierra Leone (n=6'228)	Tanzania (n=6'021)	Uganda (n=6'033)	Overall (n=24'282)	p-value
**GAD-7 ≥8, yes=1**	*(n=6'000)*	*(n=6'217)*	*(n=6'021)*	*(n=6'033)*	(n=24'270)	
**Proportion**	41.5%	19.3%	11.3%	24.6%	24.1%	0.000
**COVID-19 Exposure, yes=1**	*(n=4'670)*	*(n=5'673)*	*(n=5'550)*	*(n=4'802)*	*(n=20'695)*	
**Proportion**	31.03%	4.4%	10.9%	29.1%	17.9%	0.000
**Moderate/severe FI, yes=1**	*(n=5'951)*	*(n=6'170)*	*(n=5'952)*	*(n=5'968)*	(n=24'041)	
**Proportion**	57.5%	80.6%	24.5%	44.02%	51.9%	0.000

Note: Values are means and standard deviations or proportions. T-tests were used for comparison of continuous variables and Pearson's χ2 tests were used for comparisons of proportions. Number of non-missing values is indicated in brackets.

Table 6: Prevalence of Generalized Anxiety Disorder (GAD), Covid-19 exposure and food insecurity (unweighted)

	Mozambique (n=6'000)	Sierra Leone (n=6'228)	Tanzania (n=6'021)	Uganda (n=6'033)	Overall (n=24'282)	p-value
	(n=5'999)	*(n=6'211)*	*(n=6'019)*	*(n=6'031)*	(n=24'260)	
**GAD-7 ≥ 5, yes=1**	65.3%	66.7%	33.8%	48.2%	53.5%	0.000
**GAD-7 ≥ 8, yes=1**	40.6%	22.2%	19.4%	20.2%	25.6%	0.000
**GAD-7 ≥ 10, yes=1**	28.5%	11.7%	8.7%	11.6%	15.1%	0.000
**GAD-7 ≥ 15, yes=1**	9.3%	0.2%	0.4%	0.1%	0.3%	0.000

Note: Values are proportions. Pearson's χ2 tests were used for comparisons of proportions. Post-stratification weights were applied to all estimates. Number of non-missing values is indicated in brackets. GAD-7 = 7-item Generalized Anxiety Disorder assessed with the Patient Health Questionnaire with thresholds at score values 5, 8, 10 and 15.

Table 7: Prevalence of Generalized Anxiety Disorder (GAD) with different thresholds (with post-stratification weights)

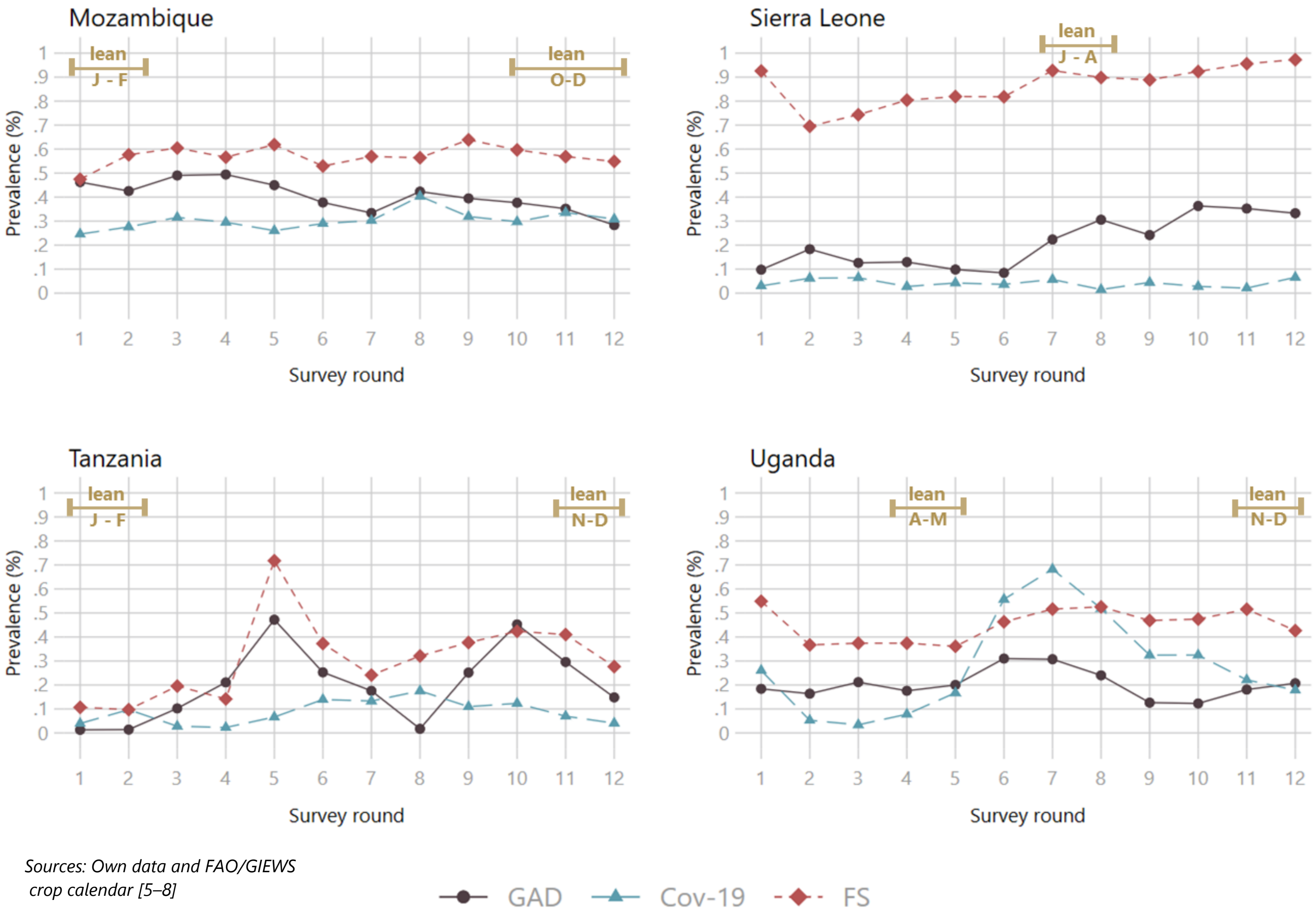

Figure 2: Prevalence over survey rounds and lean months by country (with post-stratification weights)

	No COVID-19 exposure *(n=16'848)*		COVID-19 exposure *(n=3'665)*	
Age group	FS *(n=8'164)*	FI *(n=8'684)*	FS *(n=1'828)*	FI *(n=1'837)*
18-25 *(n=3375)*	10.2%	28.8%	18.5%	27.6%
26-35 *(n=9371)*	13.7%	29.9%	20.2%	48.2%
36-45 *(n=7117)*	12.9%	29.7%	21.6%	46.6%
46-55 *(n=2917)*	16.4%	29.9%	20.5%	36.7%
56-65 *(n=1167)*	9.0%	22.7%	14.1%	52.1%
>65 *(n=313)*	8.4%	30.6%	16.8%	44.6%

Note: Values are proportions. Post-stratification weights were applied to all estimates. Number of missing observations: age group (n=10), Covid-19 exposure (n=3'576), FI (n=230), GAD (n=0)

Table 8: GAD prevalence by Covid-19 exposure, food insecurity and age group

	No COVID-19 exposure *(n=16'848)*		COVID-19 exposure *(n=3'665)*	
Age group	FS *(n=8'164)*	FI *(n=8'684)*	FS *(n=1'828)*	FI *(n=1'837)*
18-25 *(n=3375)*	10.2%	28.8%	18.5%	27.6%
26-35 *(n=9371)*	13.7%	29.9%	20.2%	48.2%
36-45 *(n=7117)*	12.9%	29.7%	21.6%	46.6%
46-55 *(n=2917)*	16.4%	29.9%	20.5%	36.7%
56-65 *(n=1167)*	9.0%	22.7%	14.1%	52.1%
>65 *(n=313)*	8.4%	30.6%	16.8%	44.6%

Note: Values are proportions. Post-stratification weights were applied to all estimates. Number of missing observations: age group (n=10), Covid-19 exposure (n=3'576), FI (n=230), GAD (n=0)

Table 9: GAD prevalence by location, gender and age group

**GAD-7 ≥8, yes=1**	Unadjusted		Adjusted	
	OR [95% CI]	P-value	OR [95% CI]	P-value
Covid exposure, yes=1	1.7 [1.4, 2.0]	0.000	1.4 [1.3, 1.6]	0.000
Moderate/severe Food Insecurity, yes=1	2.9 [2.4, 3.4]	0.000	3.1 [2.6, 3.7]	0.000
Female respondent, yes=1			1.2 [1.0, 1.3]	0.059
Age of respondent			1.0 [1.0, 1.0]	0.260
Education of respondent in years			1.0 [1.0, 1.0]	0.777
Married, yes=1			0.9 [0.7, 1.0]	0.098
Number of dependents over 60			1.0 [0.9, 1.1]	0.998
Number of dependents under 18			1.1 [1.1, 1.1]	0.000
SES index			0.8 [0.8, 0.9]	0.000
Rural household, yes=1			1.0 [0.9, 1.1]	0.961
Country = Mozambique (base category)				
Country = Sierra Leone			0.2 [0.1, 0.2]	0.000
Country = Tanzania			0.2 [0.0, 0.6]	0.010
Country = Uganda			0.1 [0.1, 0.1]	0.000
Constant	0.1 [0.1, 0.2]	0.000	0.2 [0.1, 0.3]	0.000
Observations	20'513		20'472	

Note: Logistic regression (odds ratios) excluding observations from Mozambique. Post-stratification weights were applied to all estimates; Adjusted model also accounts for region, and survey round. GAD = Generalized Anxiety Disorder

Table 10: Logistic regression (specifying country effects)

**GAD-7 ≥8, yes=1**	Unadjusted		Adjusted	
	OR [95% CI]	P-value	OR [95% CI]	P-value
COVID-19 exposure, yes=1	1.2 [1.0, 1.6]	0.104	1.2 [1.0, 1.6]	0.103
Moderate/severe Food Insecurity, yes=1	3.1 [2.4, 4.0]	0.000	4.4 [3.3, 5.7]	0.000
Female respondent, yes=1			1.0 [0.8, 1.2]	0.944
Age of respondent			1.0 [1.0, 1.0]	0.003
Education of respondent in years			1.0 [1.0, 1.0]	0.586
Married, yes=1			0.8 [0.7, 1.1]	0.125
Number of dependents over 60			0.9 [0.8, 1.1]	0.347
Number of dependents under 18			1.2 [1.1, 1.2]	0.000
SES index			0.7 [0.6, 0.8]	0.000
Rural household, yes=1			1.0 [0.8, 1.2]	0.734
Constant	0.1 [0.1, 0.1]	0.000	0.0 [0.0, 0.0]	0.000
Observations	21’871		21’830	
Observations (sub-population)	15’872		15’831	

Note: Logistic regression (odds ratios) excluding observations from Mozambique. Post-stratification weights were applied to all estimates; Adjusted model also accounts for region, country and survey round. GAD = Generalized Anxiety Disorder

Table 11: Logistic regression (excluding observations from Mozambique)

REFERENCES

1 Spitzer RL, Kroenke K, Williams, et al. A Brief Measure for Assessing Generalized Anxiety Disorder: The GAD-7. *Arch Intern Med* 2006;166(10):1092–97 (accessed 18 Nov 2022).

2 Plummer F, Manea L, Trepel D, et al. Screening for anxiety disorders with the GAD-7 and GAD-2: a systematic review and diagnostic metaanalysis. *Gen Hosp Psychiatry* 2016;39:24–31. doi:10.1016/j.genhosppsych.2015.11.005 [published Online First: 18 November 2015].

3 Food and Agriculture Organization of the United Nations (FAO). Methods for estimating comparable prevalence rates of food insecurity experienced by adults throughout the world. Rome 2016.

4 Vyas S, Kumaranayake L. Constructing socio-economic status indices: how to use principal components analysis. *Health Policy and Planning* 2006;21:459–68 (accessed 28 Oct 2022).

5 Food and Agriculture Organization of the United Nations (FAO). FAO GIEWS Country Brief on United Republic of Tanzania - 2021. Available at: https://www.fao.org/giews/countrybrief/country.jsp?code=TZA Accessed February 24, 2023.

6 Food and Agriculture Organization of the United Nations (FAO). FAO GIEWS Country Brief on Sierra Leone - 2022. Available at: https://www.fao.org/giews/countrybrief/country.jsp?code=SLE Accessed February 24, 2023.

7 Food and Agriculture Organization of the United Nations (FAO). FAO GIEWS Country Brief on Uganda - 2022. Available at: https://www.fao.org/giews/countrybrief/country.jsp?code=UGA Accessed February 24, 2023.

8 Food and Agriculture Organization of the United Nations (FAO). FAO GIEWS Country Brief on Mozambique - 2023. Available at: https://www.fao.org/giews/countrybrief/country.jsp?code=MOZ Accessed February 24, 2023.
